# Extreme decay of meteoric beryllium-10 as a proxy for persistent aridity

**DOI:** 10.1038/srep17813

**Published:** 2015-12-09

**Authors:** Rachel D. Valletta, Jane K. Willenbring, Adam R. Lewis, Allan C. Ashworth, Marc Caffee

**Affiliations:** 1 Department of Earth and Environmental Science, University of Pennsylvania, Philadelphia, Pennsylvania 19104, USA; 2 Department of Geosciences, North Dakota State University, Fargo, North Dakota 58108, USA; 3 Department of Physics and Astronomy, PRIME Lab, Purdue University, West Lafayette, Indiana 47907, USA; 4 Department of Earth, Atmospheric, and Planetary Sciences, Purdue University, West Lafayette, Indiana 47907, USA

## Abstract

The modern Antarctic Dry Valleys are locked in a hyper-arid, polar climate that enables the East Antarctic Ice Sheet (EAIS) to remain stable, frozen to underlying bedrock. The duration of these dry, cold conditions is a critical prerequisite when modeling the long-term mass balance of the EAIS during past warm climates and is best examined using terrestrial paleoclimatic proxies. Unfortunately, deposits containing such proxies are extremely rare and often difficult to date. Here, we apply a unique dating approach to tundra deposits using concentrations of meteoric beryllium-10 (^10^Be) adhered to paleolake sediments from the Friis Hills, central Dry Valleys. We show that lake sediments were emplaced between 14–17.5 My and have remained untouched by meteoric waters since that time. Our results support the notion that the onset of Dry Valleys aridification occurred ~14 My, precluding the possibility of EAIS collapse during Pliocene warming events. Lake fossils indicate that >14 My ago the Dry Valleys hosted a moist tundra that flourished in elevated atmospheric CO_2_ (>400 ppm). Thus, Dry Valleys tundra deposits record regional climatic transitions that affect EAIS mass balance, and, in a global paleoclimatic context, these deposits demonstrate how warming induced by 400 ppm CO_2_ manifests at high latitudes.

The long-standing dispute concerning the stability of the East Antarctic Ice Sheet (EAIS) calls into question its susceptibility to collapse throughout Neogene climate changes^1^. Two opposing views pervade the literature: the “dynamic” hypothesis posits that the EAIS underwent major retraction during mild Pliocene warming events, reducing to as much as two-thirds of its present size^2^,^3^,^4^, while the opposing “stable” hypothesis argues the EAIS has been largely frozen to its bed since ~14 My and has undergone only minimal, peripheral melting during Pliocene warming^5^,^6^,^7^,^8^. The dynamic theory - if correct - would imply drastic Antarctic ice mass loss and resultant sea-level rise (tens of meters) under atmospheric temperatures and CO_2_ concentrations that were only modestly greater than today (2–3 °C and 350–450 ppmv)^9^,^10^ and that are projected within the coming century^11^. With such great implications, there remains a need to expand the number of geological datasets that bear on the EAIS’s behavior through time.

Central to the dynamic/stable controversy is the timing of the onset of polar aridity, which limits substantial ice mass loss to only sublimation and is a key factor in determining long term EAIS mass balance^12^. The hyper-arid polar conditions of the Dry Valleys have protected inland sites from alteration due to weathering via precipitation or ice melt since the mid-Miocene. As such, these relict landscapes have the potential to record the inception of polar aridity and critically comment on the dynamic/stable debate, but targeted sampling locations that contain paleoclimatic proxies are uncommon and difficult to directly date.

We present a rare, continuous record of climate change contained within the innermost, highest elevation zone of the Dry Valleys. The Friis Hills, Taylor Valley (800 m above sea level) contains a thick (14 m) series of stacked glacial drifts found interbedded with silty paleolacustrine sediments. These sediments contain a diverse fossil assemblage now extinct in Antarctica including *Nothofagus* (southern beech) wood and leaves^13^. Although brine lakes commonly exist alongside and under Antarctic glaciers under the modern climatic regime^14^, the fossils within Paleolake Friis sediments were likely deposited in a semi-permanent proglacial lake on wet, freshwater tundra. Because modern climatic conditions at the Friis Hills are extremely cold (average annual temperature: −22 °C) and arid (lows measured <16% relative humidity)^15^, these deposits must archive a period of warmer and wetter climatic conditions. Directly dating these sediments becomes necessary to resolve when tundra-like conditions last prevailed in the upper, inner Dry Valleys.

## Meteoric beryllium-10 as an age indicator

To provide chronologic control for the lake sediments we utilize beryllium-10 (^10^Be) as an isotopic tracer. Cosmic-ray-produced (cosmogenic) ^10^Be forms in the atmosphere when high-energy neutrons from secondary cosmic rays spall nitrogen and oxygen atoms. This ^10^Be, denoted meteoric ^10^Be, exists in the form of ^10^BeO and ^10^Be(OH)_2_ in the atmosphere and quickly adheres to atmospheric aerosols (primarily sulfates)^16^. The ^10^Be-bearing aerosols are then delivered to the Earth’s surface through wet (rain) or dry (dust) deposition. Through continued deposition, meteoric ^10^Be will accumulate at the surface and at depth, as ^10^Be moves into the soil column via infiltration and clay illuviation^17^.

## Sediment age model

Concentrations of meteoric ^10^Be adhered to Paleolake Friis sediments are used to model a minimum age of paleolacustrine deposition. Lebatard *et al*. (ref.^17^ first demonstrated that it is possible to date ancient terrestrial deposits with meteoric ^10^Be if, once buried, sediments remain a closed system. One way to achieve this prerequisite is if meteoric waters do not infiltrate the subsurface. When these conditions are met, the measured ^10^Be reflects the initial inventory that was present at the time of burial, [^10^Be]_initial_, which is only altered by decay. A hyper-arid climate in the Dry Valleys provides the conditions needed for a closed ^10^Be system, allowing the use of meteoric ^10^Be as a chronometer.

To model sediment age, we first determine a range of potential [^10^Be]_initial_. This is possible if we model lake sediments as soil surface sediments that have reached equilibrium between ^10^Be gain (via deposition) and loss (via erosion and decay). Solving Willenbring and von Blanckenburg’s equation for steady state erosion rate (ref.^16^, Eq. 21) we estimate a likely range of [^10^Be]_initial_ that was accumulated before burial:


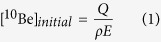


where Q is flux of ^10^Be to the Earth’s surface (atoms cm^−2^ y^−1^), ρ is soil density (1.57 g cm^−3^) and E is erosion rate (cm y^−1^). We use the ^10^Be flux calculated for Table Mountain (3.4 × 10^3^ atoms cm^−2^ y^−1^)^18^, a nearby location that is a suitable representative analog of Friis Hills because comparable arid, windy conditions disallow accumulation of atmospheric aerosols on the earth’s surface. To estimate E, we use a range of plausible erosion and total denudation rates obtained independently throughout the Dry Valleys on bedrock and regolith material (Supplementary Table S1).

Once the lake sediments were buried, the [^10^Be]_initial_ began to decay to their current concentration. To determine how long this took, we solve the radioactive decay equation for time, *t*:


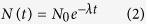


where N(t) is the measured [^10^Be] in buried lake sediments (atoms g^−1^), N_0_ is [^10^Be]_initial_ (atoms g^−1^) determined using Eq. 1 is the ^10^Be decay constant (
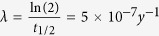
). Thus, solving Eq. 2 with a range of [^10^Be]_initial_ values yields the time range during which Paleolake Friis sediments were emplaced.

Central to our approach are measurement capabilities. The ^10^Be concentrations are measured using an accelerator mass spectrometer (AMS); the detection sensitivity is ~10^4^ atoms g^−1^. Given the half-life of ^10^Be (t_1/2_ = 1.387 My)^19^, the detection limit corresponds to a maximum age of ~14 My. That is, assuming no [^10^Be] in the buried lake sediment is lost to erosion, an AMS measurement of [^10^Be] within error of ~10^4^ atoms g^−1^ indicates a lake sediment age of at least 14 My.

## Results

**Figure 1:**
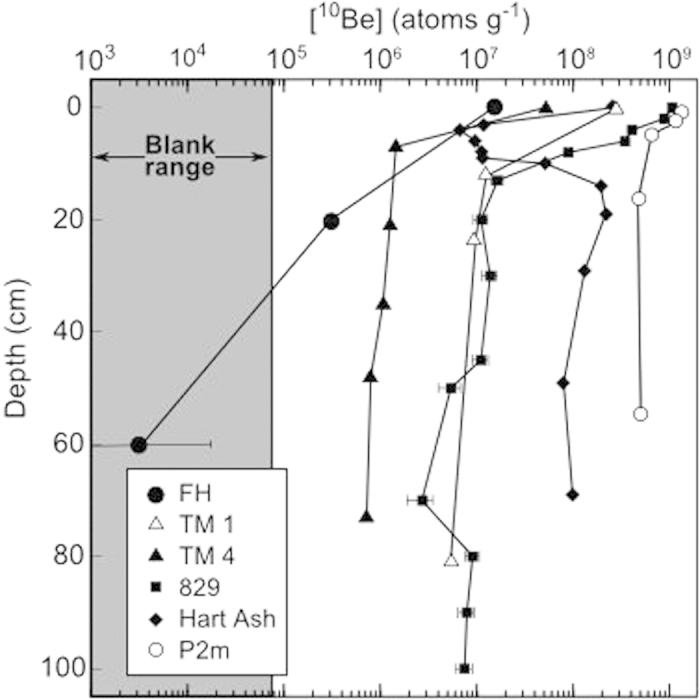
Meteoric [^10^Be] measured in shallow Dry Valleys sediments: FH (Friis Hills, this study’s Pit 1); TM 1, TM 4 (corrected for *in-situ*^10^Be contamination)^18^; 829^20^; Hart Ash (and its underlying paleosol)^21^; P2m^22^. Gray shading indicates measured [^10^Be] and associated error in the chemical blank. Results from Pit 2 register [^10^Be] <0 atoms g^−1^ and, as such, cannot be plotted on log scale.

Three samples collected at or below 26 cm depth at the Friis Hills fall below or within the 1-σ uncertainty of “blank” samples (Table 1). These measured concentrations approach the analytical limit of AMS (^10^Be/^9^Be ≈ 9 × 10^−16^) and chemical extraction process (ranging from ^10^Be/^9^Be ≈ 1 × 10^−15^ to 5 × 10^−15^). We note that other publications measure concentrations at the surface and at depth up to two and six orders of magnitude greater, respectively^19^,^21^,^22^,^23^; see Fig. 1. Higher concentrations may simply be a reflection of younger surfaces. The most comparable measurements made elsewhere are from Table Mountain (Fig. 1, profile TM4). These data have been corrected for contamination from *in situ*^10^Be. While meteoric ^10^Be is adsorbed to the outside of clay minerals, *in situ*^10^Be is produced and contained within the mineral structure itself. As Dickinson *et al*. (ref.^20^ note, *in situ* concentrations are commonly <1% of the meteoric ^10^Be concentrations, but because of the great age of Dry Valleys sediments these two fractions may be of the same magnitude. *In situ*^10^Be concentrations are most likely liberated via partial decomposition of the clay mineral due to an aggressive leaching solution. By correcting for this contamination, the authors constrain the ^10^Be flux value (Q) that we use to model the [^10^Be]_initial_ range.

**Figure 2:**
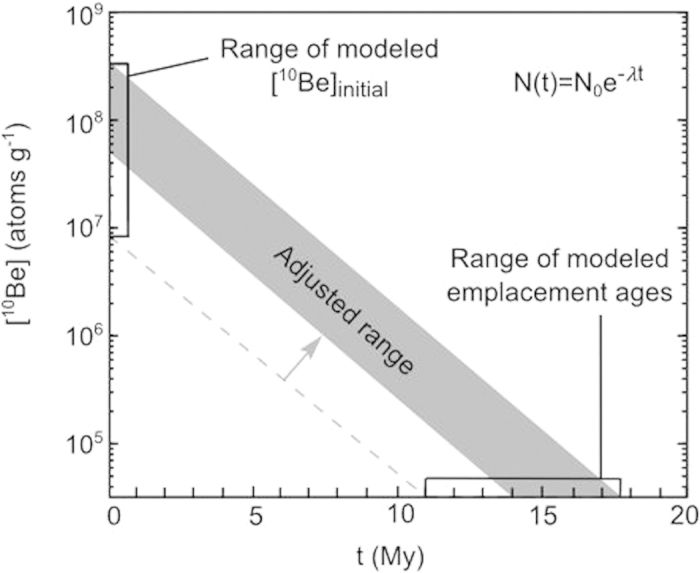
Using the radioactive decay equation, the decay of [^10^Be]_initial_ to the modern “blank” concentration (3.48 × 10^4^ ± 3.48 × 10^4^ atoms g^−1^) corresponds to a sediment emplacement age of 11.0−17.5 My. The AMS detection sensitivity of ~10^4^ atoms g^−1^ corresponds to ~14 My resulting in an adjusted age range of 14.0–17.5 My.

The modeled [^10^Be]_initial_ range is 0.83 to 22 × 10^7^ atoms g^−1^ (Supplementary Table S1). We compile a database of [^10^Be] measurements from modern and ancient lake sediments and find that our estimates are well within the range of published values (Supplementary Table S2). To determine when buried lake sediments were emplaced, we solve Eq. 2 using this [^10^Be]_initial_ range and N(t) = 3.48 × 10^4^ ± 3.48 × 10^4^ atoms g^−1^, the concentration of the chemical blank used to represent buried lake sediments, to produce a range of 11.0–17.5 My; see Fig. 2. Based on AMS measurement capabilities, lake sediments containing [^10^Be] within error of the chemical blank are at least 14 My; see above. Accordingly, we raise the lower age limit from 11 My to 14 My. The upper limit of 17.5 My is in agreement with a 19.76 ± 0.11 My (^40^Ar/^39^Ar dated) ash that lies stratigraphically below sampling Pit 1 to the east^23^. The final adjusted age range for the emplacement of Paleolake Friis sediments is 14.0–17.5 My.

### Middle Miocene climatic transitions & Paleolake Friis emplacement

Lake sediments’ age range spans the Middle Miocene Climatic Optimum (MMCO) ~15–17 My. This period is characterized by increases in global marine and terrestrial temperatures and reduced global ice coverage as indicated by marine stable isotope records^24^. In the Ross Sea region, abundant evidence for increased temperatures during the MMCO is well documented in the ANDRILL 2A core, including lithostratigraphic^25^, palynological^26^ and leaf wax abundance^27^ studies. These studies recognize periods of a retracted EAIS margin, decreased sea ice coverage, increased precipitation along the Ross Sea coastline, and a proliferation of vegetation. Definitive terrestrial evidence of the MMCO is found in high altitude tills deposited by wet-based ice^28^ and in preserved lake fossils^8^, but is otherwise sparse.

The Middle Miocene Climate Transition (MMCT) followed the MMCO at ~14 My. It is marked most notably by ice sheet expansion accompanied by a rapid, 8 °C cooling on land (14.07–13.85 My)^8^ and 6–7 °C cooling in the southern Pacific ocean (14.2-13.8 My)^29^. Marine depositional and terrestrial erosional features record this expansion in thick offshore Middle Miocene units in the Ross Sea^30^, in the cross-cutting bedrock channels of the Labyrinth, Wright Valley^31^, and in the Friis Hills themselves, where glacial expansion and down-cutting likely formed the near-modern surface^23^. A synchronized transition to arid conditions is recorded in volcanic ashes in nearby Olympus^28^ and western Asgard Ranges^5^,^6^. Workers note that ashfalls infill sand-wedge troughs, which form only in cold/dry conditions, contain glass shards, and lack evidence of cryoturbation or clay formation. This pristine preservation indicates no presence of surface moisture or chemical weathering since the time of ash emplacement. The oldest, unaltered ash deposits in the Dry Valleys indicate that other parts of the region have experienced uninterrupted polar desert climate since ~15 My^5^; our results expand this zone.

**Figure 3:**
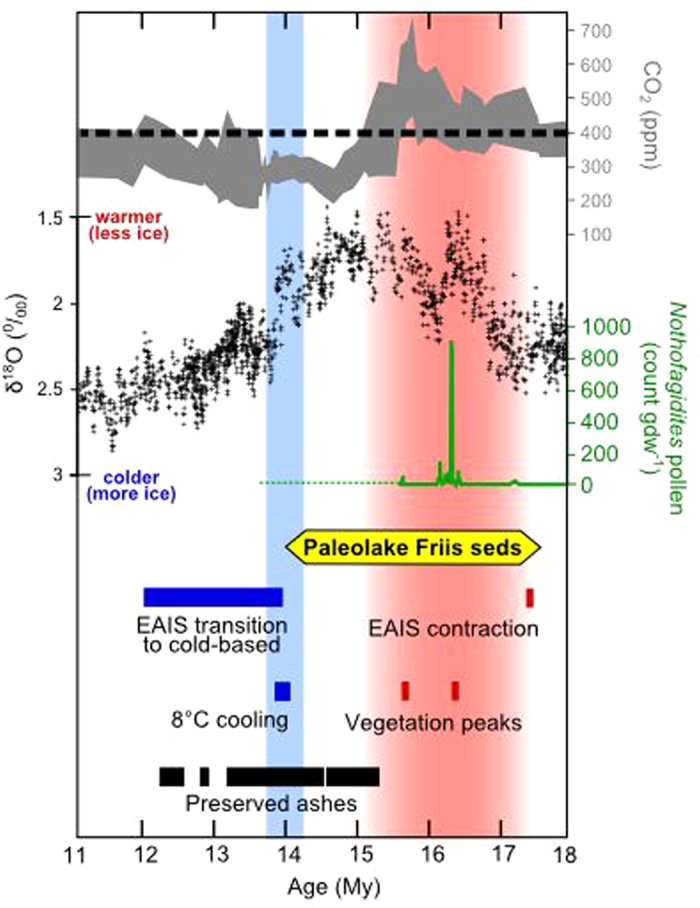
Modeled age range of Paleolake Friis sediments (14.0 to 17.5 My) and global and regional paleoclimatic indicators throughout the middle Miocene. In red: the MMCO defined globally ~15–17 My^24^ and regionally 15.4–17.6 My^25^. In blue: the MMCT, 13.8–14.2 My^29^. Labeled events include: the onset of EAIS contraction, 17.21–17.49 My^35^; peaks in vegetation expansion (including terrestrial tundra taxa and marine and freshwater algae species) centered on 15.7 and 16.4 My^27^; the range of ages of pristinely preserved ashes (measurement error included in bar width)^5^,^6^,^28^; 8 °C cooling on Antarctica^8^; EAIS transition to cold-based 12–14 My prior to major expansion at ~12 My^8^,^24^. Black crosses: global δ^18^O record (^0^/_00_), five point smooth^24^. Grey shading: global atmospheric CO_2_ (ppm) reconstruction, 5 point smooth^9^. Green line: *Nothofagidites* (type *Nothofagus fusca*) pollen abundance (count per grams dry weight, gdw^−1^) measured in AND-2A core^27^. The pollen abundance peak ~16.4 My is further evidence that *Nothofagus*, those leaf fossils in Paleolake Friis sediments, existed on Antarctica during the MMCO. The dotted green line represents a sedimentary hiatus in the AND-2A core that is attributed to ice sheet growth.

Based on the abundant evidence for warmer global and regional temperatures ~15–17 My, we suggest that Paleolake Friis sediments were likely emplaced during the MMCO (Fig. 3). At their warmest, terrestrial summer temperatures reached as high as 10 °C^26^, great enough to support a wet tundra environment in which fossils like *Nothofagus* thrived [ref.^4^ associated this species with mean summer temperatures of ~5 °C]. Warmer temperatures coincide with increases in global CO_2_ reconstructions. According to Royer’s data compilation^9^ the MMCO is arguably the last time global CO_2_ remained >400 ppm for several million years, making CO_2_ a possible driver of EAIS retraction and terrestrial plant proliferation at this time. The linkages between global CO_2_ concentrations, ice volume and vegetation during the MMCO have proven challenging to model, but these simulations are valuable towards our understanding of future climate and require improvement. A notable model deficiency is the lack of reliable temperature proxy data, particularly at high latitudes (e.g. ref.^32^). The Paleolake Friis deposits, along with those described in Lewis *et al*. (ref.^8^), represent the southernmost terrestrial deposits, and highest latitude deposits overall, available for middle Miocene paleoclimatic reconstructions. These records should be incorporated as constraints when modeling the MMCO; they are especially useful in reconstructing Equator to pole temperature gradients.

Following their deposition, Paleolake Friis sediments entered a closed system, one that did not receive meteoric ^10^Be in surface waters via ice melt or precipitation. This closed system is maintained if plunging temperatures of the MMCT ~14 My were accompanied by an onset of extreme aridity. The lack of ^10^Be in lake sediments indicates persistent polar aridity was established in the inner Dry Valleys by at least this time, contradicting the notion of large-scale EAIS collapse during the Pliocene.

## Methods

### Treating paleolake sediments

During the austral summer of 2008, five samples for meteoric ^10^Be dating were collected from silty paleolacustrine sediments exposed on a hillside within the Friis Hills stacked tills. Samples were prepared at the University of Pennsylvania Cosmogenic Isotope Lab following protocol for adhered meteoric ^10^Be extraction, including a 0.5 M HCl agitated leach and a 1 M hydroxylamine hydrochloride (NH_2_OH•HCl) leach in an ultrasonic bath^33^. Following ^9^Be spike addition (GFZ German Research Centre for Geosciences “Phenakite” standard, ^10^Be/^9^Be_spike_ = 10^−16^) and ion exchange chromatography, samples were oxidized over open flame, packed with Nb powder into cathode targets, and sent to the Purdue PRIME Lab for AMS measurement of ^10^Be/^9^Be.

### Error assessment

The overall range of erosion rates reported in the literature is 0.1–2.6 m My^−1^ corresponding to an overall range of [^10^Be]_initial_ of 0.83–22 × 10^7^ atoms g^−1^. Not all published erosion rates are reported with associated errors and cannot be recalculated because in most cases erosion rate error distributions, ^9^Be carrier spike, and/or assumed ^10^Be/^9^Be spike ratio were not reported. These missing data prohibit the inclusion of simple error propagation in our age model. Nevertheless, to assess the impact of error on our age estimates we apply a commonly reported error value of 10% to the upper and lower erosion rate estimates. This implementation results in marginally different lake sediment age estimates (10.7–17.7 My). Thus, incorporating erosion rate error does not affect our overall thesis that sediments were emplaced during the MMCO.

### Choosing a flux value, Q

The proper choice of a flux value, Q, is critical for constraining the emplacement age of Friis Hills sediments. We have chosen a relatively low flux value of 3.4 × 10^3^ atoms cm^−2^ y^−1^ because it was quantified from a nearby location with extremely similar climatic and erosional conditions^19^. Other applications of meteoric ^10^Be dating in the Dry Valleys have instead used a higher flux value that was determined from ^10^Be accumulated in the Taylor Dome ice core (1.3 × 10^5^ atoms g^−1^ y^−1^)^34^. Using this greater flux value for Paleolake Friis sediments yields a much older emplacement age of 18.2–24.8 My. This range exceeds the underlying ash’s age of 19.76 My^12^. As such, we regard the Taylor Dome flux value as unreasonably high for the Friis Hills location.

## Additional Information

**How to cite this article**: Valletta, R. D. *et al*. Extreme decay of meteoric beryllium-10 as a proxy for persistent aridity. *Sci. Rep*. **5**, 17813; doi: 10.1038/srep17813 (2015).

## Supplementary Material

Supplementary Information

## Figures and Tables

**Table 1:** Meteoric ^10^Be adhered to Paleolake Friis sediments.

Sample	Sampledepth(cm)	AMS Be 10/9(10^−14^ atomsatoms^−1^)	Error(10^−14^ atomsatoms^−1^)	[^10^Be] (10^5^atoms g^−1^)	1-σ error(10^5^ atomsg^−1^)
Friis Hills Pit 1					
ANT-08-FH-03	0	110.0	1.8	150.0	3.0
ANT-08-FH-02	20	2.4	0.24	3.1	0.36
ANT-08-FH-01	60	0.3	0.06	0.03	0.1
Friis Hills Pit 2					
ANT-08-FH-05	26	0.1	0.04	−0.1	0.1
ANT-08-FH-04	30	0.20	0.11	−0.054	0.18
Chemical blanks					
1	N.A.	0.2	0.08	0.3	0.3
2	N.A.	0.2	0.09	0.3	0.3
