# Supplementary material for: Extreme decay of meteoric beryllium-10 as a proxy for persistent aridity
Source: Sci Rep. 2015 Dec 9;5:17813. doi: 10.1038/srep17813 (PMC4673429; doi:10.1038/srep17813)
Supplement: Supplementary Information [file srep17813-s1.pdf]

Extreme decay of meteoric beryllium-10 as a proxy for persistent aridity:

Supplementary Document

Rachel D. Valletta[1*], Jane K. Willenbring[1], Adam R. Lewis[2], Allan C. Ashworth[2], and Marc Caffee[3,4]

Supplementary Table S1: Erosion rates used to estimate Paleolake Friis sediment age			
**Reference**	**Material**	**Min-Max E ($m\ My^{-1}$)**	**Min-Max $[^{10}Be]_{initial}$ (atoms g^{-1} x 10^7)***
36	Sandstone boulder	0.32-1.31	1.65-6.77
37	Sandstone, granite boulders	0.133-1.02	2.12-16.28
38	Regolith	2.1	1.03
39	Regolith	0.19-2.6	0.83-11.40
18	Diamicton, sandstone	0.1-0.33	6.56-21.66
	**Overall range**	**0.1-2.6**	**0.83-22.0**
	**Predicted age range (My)** †	**11.0-17.5**	

*Calculated using Eq. 1 and parameters as defined in text.
† Calculated using Eq. 2

Supplementary Table S2: Concentration ranges of meteoric ^{10}Be adhered to lake sediments worldwide

Location	Sample type	Minimum [^{10}Be] (atoms g^{-1})	Maximum [^{10}Be] (atoms g^{-1})	Reference
Lake Lisan (Dead Sea), Negev Desert, Israel	Bulk lake bottom sediment	0.78±0.03 x 10^8	1.64±0.07 x 10^8	40
Lake Lehmilampi, Finland	Cored lake bottom sediment	2.1 x 10^8	1.76 x 10^9	41
Anderson Pond, Tennessee, USA	Cored lake bottom sediment	2.16±0.13 x 10^9	2.90±0.13 x 10^9	42
Lake Baikal, Russia	Cored lake bottom sediment	5.07±0.39 x 10^8	1.13±0.05 x 10^9	43
Lake Baikal, Russia	Cored lake bottom sediment	0.5 x 10^9	1.5 x 10^9	44
Lake Mega-Chad, North Africa	Paleolacustrine sediments	2.38±0.25 x 10^6	8.59±0.35 x 10^7	17
Hillpiece Bog, Tristan da Cunha	Lacustrine sediments	7.02±0.379 x 10^8	1.75±0.98 x 10^9	45
Union Lake, NJ. USA	Bulk lake bottom sediment	1.20±0.60 x 10^7	2.60±0.30 x 10^{10}	46
Lake Keilambete, Australia	Bulk lake bottom sediment	0.76 x 10^9	2.31 x 10^9	47
Lake Windermere, England	Bulk lake bottom sediment	1.11 x 10^9	1.6 x 10^9	47
Lake Zurich, Switzerland	Sediment trap sediments	4.96±0.32 x 10^7	2.54±0.20 x 10^8	48
Mono Lake, CA, USA	Bulk lake bottom sediment	0.7 x 10^8	4.1 x 10^8	49
**Overall average† (atoms g^{-1})**		2.38x10^6	2.90x10^9	
**[^{10}Be]$_{initial}$ estimates at Friis Hills**		8.3x10^6	2.2x10^8	

* Measurements initially reported in dpm kg^{-1}. Converted to atoms g^{-1} using the following: dpm (decays per minute) = A (radioactive activity) and A=λN, where N=atoms of ^{10}Be and λ=5.0x10^{-7} y^{-1}.

† Not all values are published with associated error. As such, overall minimum and maximum [^{10}Be] are calculated without reported error. Where an external age estimator was provided, the effect of decay correction was calculated. Maximum decay corrected concentrations differ < 3% from non-corrected values, and are not included in the overall average.

Supplementary References

36. Nishiizumi, K., Kohl, C. P., Arnold, J. R., Klein, J., & Middleton, R. Cosmic ray produced ^{10}Be and ^{26}Al in Antarctic rocks: Exposure and erosion history. *Earth Planet. Sc. Lett.* **104**, 440–454 (1991).

37. Summerfield, M.A., et al. Long-term rates of denudation in the Dry Valleys, Transantarctic Mountains, southern Victoria Land, Antarctica based on in-situ-produced cosmogenic ^{21}Ne. *Geomorphology* **27**, 113-129 (1999).

38. Putkonen, J., Balco, G., & Morgan, D. Slow regolith degradation without creep determined by cosmogenic nuclide measurements in Arena Valley, Antarctica. *Quat. Res.* **69**, 242-249 (2008).

39. Morgan, D., Putkonen, J., Balco, G., & Stone, J. Quantifying regolith erosion rates with cosmogenic nuclides ^{10}Be and ^{26}Al in the McMurdo Dry Valleys, Antarctica. *J. Geophys. Res.* **115**, F0307 (2010).

40. Belmaker, R., Lazar, B., Tepelyakov, N., Stein, M., & Beer, J., ^{10}Be in Lake Lisan sediments-A proxy for production or climate? *Earth Planet. Sc. Lett.* **269**, 448-457 (2008).

41. Berggren, A.-M., Aldahan, A., Possnert, G., Haltia-Hovi, E., & Saarinen, T. Linking ice sheet and lake sediment archives of ^{10}Be, 1468-1980 CE. *Nucl. Instrum. Meth. B* **294**, 524-529 (2013).

42. Brown, T.A., Nelson, D.E., Southon, J.R., & Vogel, J.S. ^{10}Be production rate variations as recorded in a mid-latitude lake sediment. *Nucl. Instrum. Meth. B* **29**, 232-237 (1987).

43. Horiuchi, K., et al. Last-glacial to post-glacial ^{10}Be fluctuations in a sediment core from the Academician Ridge, Lake Baikal. *Geophys. Res. Lett.* **26**, 1047-1050 (1999).

44. Horiuchi, K., et al. Climate-induced fluctuations of ^{10}Be concentration in Lake Baikal sediments. *Nucl. Instrum. Meth. B* **172**, 562-567 (2000).

45. Ljung, K., Björck, S., Muscheler, R., Beer, J., & Kubik, P. W. Variable ^{10}Be fluxes in lacustrine sediments from Tristan da Cunha, South Atlantic: a solar record? *Quat. Sci. Rev.* **26**, 829-835 (2007).

46. Lundberg, L., et al. ^{10}Be and Be in the Maurice River-Union lake system of southern New Jersey. *J. Geophys. Res.* **88**, 4498-4504 (1983).

47. Raisbeck, G.M., et al. ^{10}Be in the environment: some recent results and their applications. *Proc. Syrup. Accel. Mass Spectrom. Argonne, II1: Argonne Natl. Lab.*, 458 (1981).

48. Schuler, C., et al. A multitracer study of radionuclides in Lake Zurich, Switzerland 1. Comparison of atmospheric and sedimentary fluxes of ^{7}Be, ^{10}Be, ^{210}Pb, ^{210}Po, and ^{137}Cs. *J. Geophys. Res.* **96**, 17051-17065 (1991).

49. Ticich, T., Lundberg, L., Pal, D.K., Smith, C.M., & Herzog, G.F. ^{10}Be contents of Mono Lake sediments: search for enhancement during a geomagnetic excursion. *Geophys. J. Int.* **87**, 487-492 (1986).
